# Impact of closed management on gastrointestinal function and mental health of Chinese university students during COVID-19

**DOI:** 10.1186/s12889-023-16145-1

**Published:** 2023-06-23

**Authors:** Kaini Wu, Yi Li, Yating Pan, Jianhao Qiu, Xiaqin Chen, Yuanping Fan, Yawei Xing, Xiaodong Zhou

**Affiliations:** Department of Gastroenterology, Digestive Disease Hospital, The First Affiliated Hospital of Nanchang University, No. 17, Yongwaizheng Street, Nanchang, 330006 Jiangxi Province China

**Keywords:** COVID-19, Gastrointestinal function, Mental health, Closed management, College students

## Abstract

**Background:**

The innovative closed management of universities may have influenced the physical and mental health of students during the fourth stage of the COVID-19 pandemic in China. The study aimed to assess the gastrointestinal and mental health status of students in this stage and to explore the possible risk factors and mechanisms to provide a reference for future school responses to similar stressful events.

**Method:**

A multicenter, cross-sectional survey was administered to 598 college students from 10 Chinese universities. The study used the 7-item Generalized Anxiety Disorder Scale (GAD-7), 9-item Patient Health Questionnaire (PHQ-9), Fear of COVID-19 Scale (FCV-19 S), and the Diagnostic Tendency of Functional Bowel Disease Scale (DT-FBD) to evaluate anxiety, depression, fear of COVID-19 and likelihood of being diagnose diagnosed with functional bowel disease (FBD), respectively.

**Results:**

A total of 516 college students completed the questionnaire. The proportions of students with more severe anxiety, more severe depression, greater fear of COVID-19, and a greater likelihood of being diagnosed with FBD were 49.8%, 57.0%, 49%, and 49%, respectively. These symptoms were significantly and positively correlated with the frequency of irregular sleep and eating (p < 0.05). Students in high-risk areas were more likely to experience anxiety and depression than students in areas with low/medium risk (odds ratio [OR] = 1.90, 95% confidence interval [CI]: 1.12–3.24, p = 0.017; OR = 2.14, 95% CI: 1.11–4.11, p = 0.022). A high likelihood of being diagnosed with FBD was positively associated with the severity of anxiety and depression symptoms and fear of COVID-19 (all p < 0.001). Moreover, mediation analysis revealed the following pathway in college students: fear of COVID-19 → depression and anxiety → poor diet → likelihood of being diagnosed with FBD.

**Conclusion:**

College students generally exhibited higher more severe anxiety and depression symptoms and psychological symptoms with a greater higher propensity likelihood of being to be diagnosed with FBD. Good lifestyle habits, especially adequate sleep and a regular diet, can alleviate these problems. In addition, appropriate psychological intervention is very important.

**Supplementary Information:**

The online version contains supplementary material available at 10.1186/s12889-023-16145-1.

## Introduction

On January 30, 2020, the World Health Organization declared the novel coronavirus disease 2019 (COVID-19) a public health emergency of international concern; as of September 30, 2022, there have been with over 600 million cases of COVID-19 and over 6 million deaths [1]. After the initial outbreak, the spread of COVID-19 in China was rapidly controlled. However, after the end of February 2022, at which time the mutant Omicron strain of COVID-19 was prevalent, outbreaks were reported in several places, one after another. Chinese experts refer to this phase as the “fourth stage” of the pandemic in China [2]. Many universities adopted partial or entire lockdown measures, restricting students from leaving campus unless necessary. Furthermore, nonstudents were not allowed to enter the campus without permission, and some schools required instructors to switch to remote learning to reduce the risk of exposure [3].

Nevertheless, studies have suggested that these strict containment measures may have adversely affected the mental health of college students during the COVID-19 pandemic. A meta-analysis showed that pandemic-induced disruption of academic routines and increased isolation among college students increased the combined prevalence of depressive, anxiety, and sleep disorders [4]. A higher number of university students experienced anxiety (of varying severity) during the COVID-19 lockdown in Malaysia [5]. Several other studies have also highlighted that COVID-19 lockdowns were associated with higher psychological symptoms [6, 7]. Unfortunately, little research has been conducted on the impact of the innovative closed management strategy adopted by Chinese universities on students. Most of the articles have focused on the mental crisis in this population, with little attention given to the characteristics of gastrointestinal disorders at this stage [8–10]. Moreover, the vast majority of articles on COVID-19 lockdown measures and gastrointestinal illness have focused on patients with gastrointestinal disorders, rather than healthy individuals, especially students [11–15].

Functional bowel disease (FBD) is chronic condition characterized by persistent and recurring bowel symptoms [16]. FBD is common worldwide, and the prevalence of irritable bowel syndrome (IBS) is 10–15% in the general population [17, 18]. The pathogenesis of FBD is still unclear [19], but numerous studies have reported that FBD may be closely related to psychological disorders, lifestyle habits, and other factors [16, 17, 20]. Several students have reported recurrent symptoms of abdominal pain, bloating, abdominal discomfort, diarrhea, or constipation during the closed management, which are similar to symptoms of FBD [21]. Therefore, we believe that closed management may have a negative effect on the gastrointestinal function of college students, and may be related to individuals’ mental health.

The purpose of this study was to evaluate the levels of intestinal dysfunction, anxiety, and depression among college students and associated risk factors, and to provide recommendations for the management of the physical and mental health of students during stressful events such as outbreaks of other infectious diseases. In addition, it was hypothesized that the adverse effects of closed management measures during the epidemic on mental health would lead to poor lifestyle habits and thus affect the gastrointestinal health of college students. We constructed a mediation model to further explore the mechanism of occurrence and provide support for the rationality and necessity of relevant policies.

## Methods

### Design and participants

China was divided into three regions (high risk, medium risk, and low or no risk), based on the daily aggregation of outbreak risk data reported by the National Health and Wellness Commission of China (recorded on 2022-04-22); these regions did not change during the questionnaire collection period. This cross-sectional study examined college students in all three risk areas. Ten colleges and universities from 9 cities nationwide were randomly selected, and students were invited to complete an online questionnaire. Subjects with the following conditions were excluded: peptic ulcer, gastroesophageal reflux disease, inflammatory bowel disease and other organic diseases of the gastrointestinal tract, psychoneurological disorders, COVID-19 infection, and continuous use of the same drug within the last two weeks. This study was approved by the Human Ethics Committee of The First Affiliated Hospital of Nanchang University. Studies involving human study participants were conducted in accordance with the Declaration of Helsinki. All involved persons provided written informed consent prior to study inclusion.

### Instruments and questionnaire

Questionnaire Star (https://www.wjx.cn/), the most commonly used online questionnaire system in China, was used to distribute the questionnaire. Two reviewers (KNW, YL) entered the obtained data to prevent errors in data entry. The questionnaire consisted of 43 questions and included three parts: (1) participant demographic data (age, sex, education level, risk region, professional category, diet, sleep status in the last two weeks, average daily exercise duration in the last two weeks, and internet use duration), (2) standardized scales, including the 7-item Generalized Anxiety Disorder scale (GAD-7), 9-item Patient Health Questionnaire (PHQ-9), and Fear of COVID-19 Scale (FCV-19 S), and (3) a custom-designed scale, the Diagnostic Tendency of Functional Bowel Disease Scale (DT-FBD).

#### The 7-item generalized anxiety disorder scale (GAD-7)

The GAD-7 is the most widely used anxiety measurement tool in clinical practice and research. According to Spitzer et al., the cutoff point is a score ≥ 5 [22]. This scale has good discriminant validity and reliability as demonstrated in several studies (Cronbach’s α > 0.9). The Chinese version of this scale has validity for estimating anxiety [22–24]. Cronbach’s α in this study was 0.930.

#### The 9-item Patient Health Questionnaire (PHQ-9)

The PHQ-9 is a self-administered scale used to measure depressive symptoms. The cutoff point was a score ≥ 7 [25]. The Chinese version of the PHQ-9 showed satisfactory reliability (Cronbach’s α of 0.854) and sensitivity in college students [26]. Cronbach’s α in the present study was 0.925.

#### The fear of COVID-19 scale (FCV-19 S)

The FCV-19 S was developed by Ahorsuet et al. [27] to assess individual’s fear of COVID-19. A 5-point Likert scale was is adoptedused, with higher scores indicating a higher level greater of fear of COVID-19. The reliability and validity of the questionnaire have been demonstrated in several countries, including China [28, 29]. In the present study, Cronbach’s α was 0.934.

#### The diagnostic tendency of functional bowel Disease Scale (DT-FBD)

The DT-FBD is a self-report scale designed to evaluate the likelihood that individuals will be diagnosed with FBD. The current widely used questionnaire for FBD is the Rome III standard questionnaire, but it is mainly used in medical institutions and has many items. To persuade more students to participate in this experiment, the DT-FBD was developed based on the Rome III criteria (and able to reflect all the symptoms of FBD as defined by the criteria) but in a simplified form. This study had good reliability and validity, with a Cronbach’s α of 0.872.

### Data analysis

Continuous variables are presented as the median and interquartile range (IQR). Categorical variables are expressed as frequencies and percentages. A binary logistic regression model was used to assess risk factors. Univariate logistic regression analysis was performed to identify significant influencing factors (p < 0.1), and multivariate analysis was conducted after controlling for possible confounding factors. Correlation analysis was performed to assess potential confounders and interactions. According to the correlation matrix, we used the DT-FBD score as the independent variable, the FCV-19 S score as the dependent variable, and the GAD-7 score, PHQ-9 score, and frequencies of irregular diet and irregular sleep within the last two weeks as mediating variables. Mplus 8.0 was used to conduct the mediation models by sampling 5000 times with the bootstrap method. The associations are reported as the odds ratios (ORs) or unstandardized coefficients and 95% confidence intervals (CIs). P values < 0.05 were considered statistically significant. Statistical analyses were performed using IBM SPSS for Windows version 26.0.

## Results

### Demographic characteristics

**Fig. 1:**
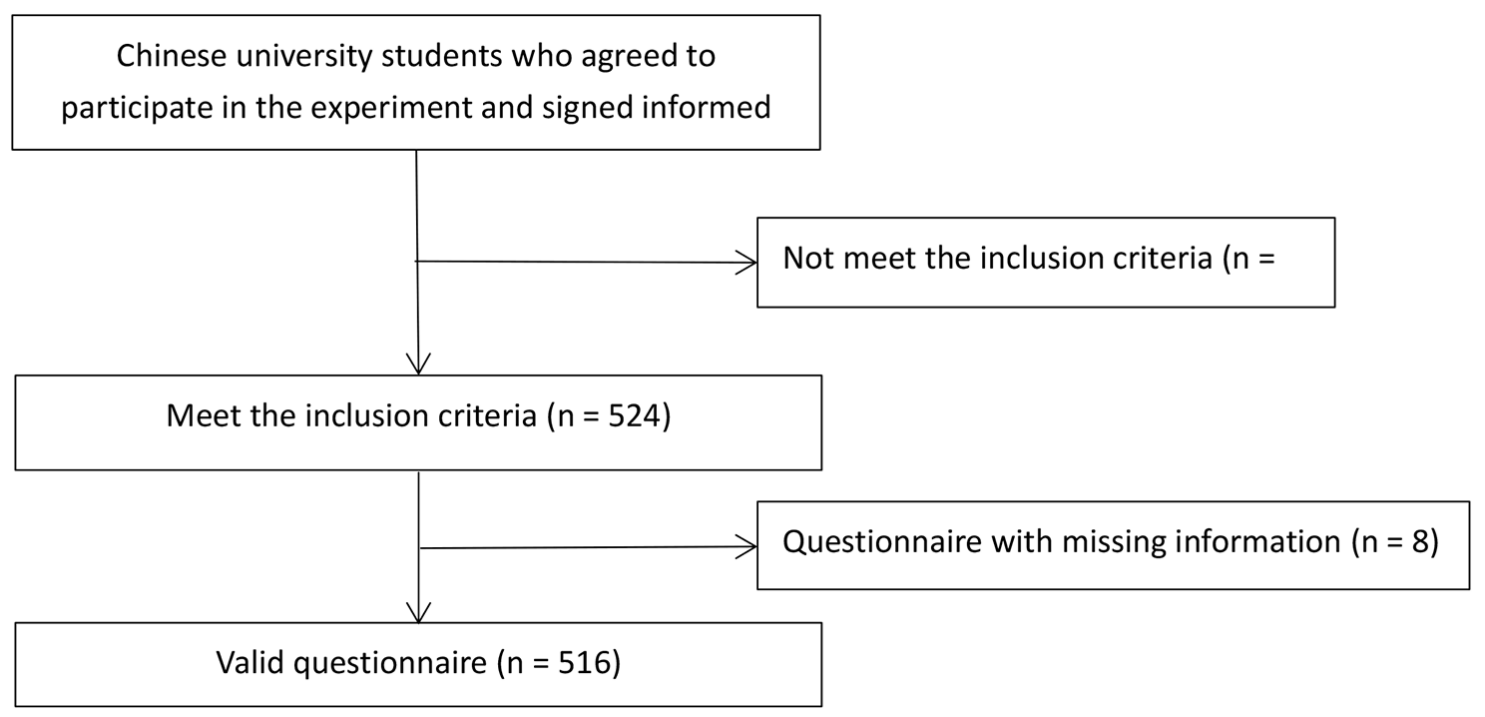
Flow chart of questionnaire survey. 598 students from 10 Chinese universities agreed to participate in the questionnaire and signed informed consent. 64 questionnaires that did not meet the inclusion criteria were excluded, and 524 questionnaires that met the inclusion criteria were obtained. 8 questionnaires with missing information were excluded, resulting in 516 valid questionnaires

From April 24 to May 7, 2022, 598 questionnaires were collected from students at 10 Chinese universities. As shown in Figs. 1 and 64 questionnaires did not meet the inclusion criteria and, 8 questionnaires had missing information; thus, 516 valid questionnaires were finally included. The demographic characteristics of the 516 students included in the study are shown in Table 1. The median age of the sample was 20 years old (IQR: 19–21), of which 41.9% (216/516) were male and 58.1% (300/516) were female. The percentages of students in low/medium and high-risk areas were 84.3% (435/516) and 15.7% (81/516), respectively.

**Table 1:** Demographic characteristics of the university students during the period of closed school management under the epidemic

Characteristics	Total n = 516
Age, M (IQR)	20 (2)
Male, n (%)	216 (41.9)
Education	
Undergraduate	436 (84.5)
Graduate	80 (15.5)
Major, n (%)	
Medicine	217 (42.1)
Natural Sciences (expect medicine)	179 (34.7)
Social Sciences	120 (23.3)
Region	
Low/medium risk	435 (84.3)
High risk	81 (15.7)
Meal, n (%)	
No	253 (49)
A few days	161 (31.2)
More than a week	54 (10.5)
Almost every day	48 (9.3)
Sleep, n (%)	
No	198 (38.4)
A few days	194 (19.4)
More than a week	69 (13.4)
Almost every day	55 (10.7)
Exercise, n (%)	
No	66 (12.8)
< 30 min	268 (51.9)
30–60 min	144 (27.9)
>60 min	38 (7.4)
Internet, n (%)	
≤ 2 h	19 (3.87)
2–5 h	152 (29.5)
>5 h	345 (66.9)
GAD-7, M (IQR)	4 (6)
PHQ-9, M (IQR)	5 (7)
FCV-19 S, M (IQR)	23 (14)
DT-FBD, M (IQR)	9 (4)

Meal, frequency of irregularity of three meals in the last two weeks; Sleep, frequency of irregular sleep in the last two weeks; Exercise, average daily exercise time in the last two weeks; Internet, average daily Internet usage in the last two weeks; GAD-7, the 7-item Generalized Anxiety Disorder Scale; PHQ-9, the 9-item Patient Health Questionnaire; FCV-19 S, the Fear of COVID-19 Scale; DT-FBD, the Diagnostic Tendency of Functional Bowel Disease Scale

Regarding the GAD-7, PHQ-9, and FCV-19 S, we calculated the proportion of participants that exceeded the cutoff values mentioned above. The median (score of 9) on the DT-FBD was used as the cutoff value for determining positive events on our custom-designed scale, with scores greater than or equal to 9 considered to indicate individuals with a higher likelihood of being diagnosed with FBD. Of the included students, 49% (254/516) had a higher likelihood of being diagnosed with FBD, 49.8% (257/516) had severe anxiety, and 57.0% (294/516) had severe depression. In addition, 49% (254/516) of the students had a high level of fear of COVID-19.

### Binary logistic regression analysis of high propensity to diagnose FBD, anxiety, depression, and the fear of COVID-19

As shown in Table 2, the likelihood of being diagnosed with FBD was related to sex and was higher in females than in males (OR = 2.29, 95% CI: 1.52–3.46, p < 0.001). In addition, greater frequencies of irregularity in diet and sleep in the last two weeks were associated with a higher likelihood of being diagnosed FBD (OR = 1.77, 95% CI: 1.37–2.28, p < 0.001 and OR = 1.95, 95% CI: 1.58–2.41, p < 0.001, respectively). This propensity was also positively associated with the severity of anxiety and depressive symptoms and the fear of COVID-19 (OR = 1.143, 95% CI: 1.08–1.21, p < 0.001; OR = 1.15, 95% CI: 1.08–1.23, p < 0.001; and OR = 1.05, 95% CI: 1.03–1.07, p < 0.001, respectively).

**Table 2:** Univariate and multivariate logistic regression analyses of high propensity to diagnose FBD among university students during the period of closed school management under the epidemic

Variable	Univariable OR (95% CI)	P-value	Multivariable OR (95% CI)	P-value
Age	1.03 (0.95–1.11)	0.531	-	-
Sex	2.06 (1.44–2.94)	< 0.001	2.29 (1.52–3.46)	< 0.001
Education	0.92 (0.57–1.49)	0.737	-	-
Major	1.13 (0.91–1.41)	0.275	-	-
Region	1.35 (0.84–2.18)	0.216	-	-
Meal	2.18 (1.76–2.70)	< 0.001	1.77 (1.37–2.28)	< 0.001
Sleep	1.92 (1.57–2.34)	< 0.001	1.95 (1.58–2.41)	< 0.001
Exercise	0.82 (0.66–1.02)	0.076	0.83 (0.66–1.03)	0.091
Internet	1.41 (1.03–1.94)	0.032	1.34 (0.97–1.86)	0.081
Anxiety	1.20 (1.15–1.26)	< 0.001	1.143 (1.08–1.21)	< 0.001
Depression	1.21 (1.16–1.26)	< 0.001	1.15 (1.08–1.23)	< 0.001
Fear of COVID-19	1.05 (1.03–1.08)	< 0.001	1.05 (1.03–1.07)	< 0.001

FBD, functional bowel disease; Meal, frequency of irregularity of three meals in the last two weeks; Sleep, frequency of irregular sleep in the last two weeks; Exercise, average daily exercise time in the last two weeks; Internet, average daily Internet usage in the last two weeks

Table 3 presents the risk factors related to the mental health of college students. Students in high-risk areas were more likely to exhibit anxiety and depressive symptoms than students in low- or moderate-risk areas (OR = 2.14, 95% CI: 1.11–4.11, p = 0.022). Furthermore, anxiety and depression levels were positively correlated with the frequency of irregular eating (OR = 1.88, 95% CI: 1.41–2.50, p < 0.001; OR = 1.80, 95% CI: 1.32–2.45, p < 0.001), the frequency of irregular sleep (OR = 1.81, 95% CI: 1.37–2.39, p < 0.001; OR = 2.50, 95% CI: 1.83–3.40, p < 0.001) and the average daily internet use of students in the last two weeks (OR = 1.44, 95% CI: 1.05–1.99, p = 0.025; OR = 1.54, 95% CI: 1.02–2.30, p = 0.038). In addition, anxiety and depression levels were found to increase as the fear of COVID-19 increased (OR = 1.10, 95% CI: 1.07–1.14, p < 0.001; OR = 1.09, 95% CI: 1.06–1.12, p < 0.001). Notably, exercise duration was associated with anxiety symptoms, with longer average daily exercise durations associated with reduced anxiety symptoms (OR = 0.73, 95% CI: 0.57–0.93, p = 0.011).

**Table 3:** Univariate and multivariate logistic regression analyses of anxiety and depression among university students during the period of closed school management under the epidemic

anxiety					depression			
Variable	Univariable OR (95% CI)	P-value	Multivariable OR (95% CI)	P-value	Univariable OR (95% CI)	P-value	Multivariable OR (95% CI)	P-value
Age	0.93 (0.85–1.01)	0.072	1.16 (0.99–1.36)	0.075	0.91 (0.84-1.00)	0.025	1.18 (0.99–1.40)	0.063
Sex	1.32 (0.93–1.87)	0.126	-	-	0.97 (0.68–1.38)	0.867	-	-
Education	0.43 (0.26–0.71)	0.001	0.31 (0.11–0.86)	0.025	0.39 (0.24–0.64)	< 0.001	0.26 (0.09–0.73)	0.011
Major	1.20 (0.96–1.50)	0.109	-	-	1.22 (0.97–1.52)	0.085	1.05 (0.78–1.41)	0.747
Region	1.35 (0.84–2.89)	0.020	1.90 (1.12–3.24)	0.017	2.28 (1.35–3.86)	0.002	2.14 (1.11–4.11)	0.022
Meal	2.69 (2.13–3.41)	< 0.001	1.88 (1.41–2.50)	< 0.001	3.06 (2.34–3.99)	< 0.001	1.80 (1.32–2.45)	< 0.001
Sleep	2.65 (2.02–3.32)	< 0.001	1.81 (1.37–2.39)	< 0.001	3.59 (2.74–4.72)	< 0.001	2.50 (1.83–3.40)	< 0.001
Exercise	0.69 (0.55-0.87)	0.001	0.73 (0.57–0.93)	0.011	0.80 (0.64–0.99)	0.045	0.83 (0.66–1.05)	0.117
Internet	1.38 (1.01–1.89)	0.045	1.44 (1.05–1.99)	0.025	1.93 (1.40–2.66)	< 0.001	1.54 (1.02–2.30)	0.038
Fear of COVID-19	1.11 (1.08–1.14)	< 0.001	1.10 (1.07–1.14)	< 0.001	1.10 (1.07–1.12)	< 0.001	1.09 (1.06–1.12)	< 0.001

Meal, frequency of irregularity of three meals in the last two weeks; Sleep, frequency of irregular sleep in the last two weeks; Exercise, average daily exercise time in the last two weeks; Internet, average daily Internet usage in the last two weeks

The fear of COVID-19 was higher in females than in males (OR = 1.46, 95% CI: 1.02–2.84, p = 0.037). As seen in Table 4, increased frequencies of irregular eating and sleep in the last two weeks were associated with higher levels of fear (OR = 1.29, 95% CI: 1.07–1.55, p = 0.009; OR = 1.34, 95% CI: 1.12–1.61, p = 0.002). The lower the average daily exercise duration in the last two weeks was, the higher the level of fear (OR = 0.74, 95% CI: 0.59–0.93, p = 0.009). Moreover, the severity of anxiety and depressive symptoms was significantly positively correlated with the fear of COVID-19 (OR = 1.11, 95% CI: 1.04–1.19, p = 0.004; OR = 1.08, 95% CI: 1.04–1.12, p < 0.001).

**Table 4:** Univariate and multivariate logistic regression analyses of the fear of COVID-19 among university students during the period of closed school management under the epidemic

Variable	Univariable OR (95% CI)	P-value	Multivariable OR (95% CI)	P-value
Age	0.90 (0.82–0.97)	0.010	0.93 (0.81–1.08)	0.332
Sex	1.44 (1.01–2.04)	0.044	1.46 (1.02–2.84)	0.037
Education	0.44 (0.27–0.73)	0.001	0.53 (0.31–0.90)	0.019
Major	1.24 (0.99–1.54)	0.061	1.18 (0.93–1.50)	0.185
Region	1.01 (0.63–1.62)	0.975	-	-
Meal	1.34 (1.12–1.62)	0.002	1.29 (1.07–1.55)	0.009
Sleep	1.33 (1.11–1.60)	0.002	1.34 (1.12–1.61)	0.002
Exercise	0.75 (0.60–0.94)	0.011	0.74 (0.59–0.93)	0.009
Internet	1.18 (0.86–1.61)	0.300	-	-
Anxiety	1.13 (1.09–1.82)	< 0.001	1.11 (1.04–1.19)	0.004
Depression	1.09 (1.05–1.13)	< 0.001	1.08 (1.04–1.12)	< 0.001

Meal, frequency of irregularity of three meals in the last two weeks; Sleep, frequency of irregular sleep in the last two weeks; Exercise, average daily exercise time in the last two weeks; Internet, average daily Internet usage in the last two weeks

### Mediation analysis

**Fig. 2:**
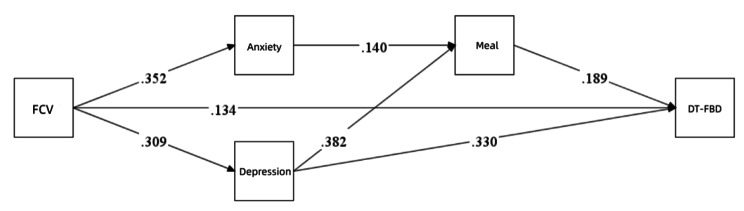
Mediation relationship model. All relationships in the figure are significant (p < 0.01). FCV, Fear of COVID-19; Meal, frequency of irregularity of three meals in the last two weeks; DT-FBD, the Diagnostic Tendency of Functional Bowel Disease. Using Mplus 8.0 for mediational modeling. Fear of COVID-19 was able to directly influence DT-FBD, in addition to indirectly influencing GI symptoms by affecting students’ mental health status (anxiety, depression), which in turn influenced eating habits

Correlation analyses were performed to investigate the correlations between all variables; the correlation matrix is shown in Table 5. Figure 2 displays the multiple chain mediation model with GAD-7 scores, PHQ-9 score, and the frequency of dietary irregularity in the last two weeks included as mediators (with standardized path coefficients). For this model, the RMSEA was 0.000, the Akaike information criterion (AIC) was 7826.861, and the BIC was 7924.521. The study revealed that FCV-19 S scores positively predicted GAD-7, PHQ-9 and DT-FBD scores (β = 0.352, SE = 0.004, p < 0.001; β = 0.309, SE = 0.005, p < 0.001; β = 0.134, SE = 0.022, p = 0.013). Importantly, the frequency of irregular diet in the last two weeks and PHQ-9 scores predicted DT-FBD scores (β = 0.189, SE = 0.216, p < 0.001; β = 0.330, SE = 0.240, p < 0.001), suggesting that irregular diet and PHQ-9 scores mediated the relationship between FCV-19 S scores and DT-FBD scores. In addition, the frequency of irregular eating in the past two weeks was positively predicted by GAD-7 and PHQ-9 scores (β = 0.140, SE = 0.074, p < 0.001; β = 0.035, SE = 0.057, p < 0.001), indicating that symptoms of anxiety and depression act as chain mediators of the relationship between the frequency of dietary irregularity and DT-FBD scores. As shown in Table 6, both the direct effect and the indirect effect of the relationship between FCV-19 S scores and DT-FBD scores were significant, with the mediating variable explaining 43.89% of the total variance. Moreover, the results of our analyses showed that the frequency of irregular eating as well as GAD-7 and PHQ-9 scores partially mediated the relationship between FCV-19 S scores and DT-FBD scores.

**Table 5:** Correlation Matrix analysis of included variables that provides a reference for mediation analysis

Variable	Age	Sex	Education	Major	Region	Meal	Sleep	Exercise	Internet	Anxiety	Depression	Fear of COVID-19	DT-FBD
Age	1.000												
Sex	0.033	1.000											
Education	0.780**	-0.016	1.000										
Major	0.006	0.140**	-0.123**	1.000									
Region	0.062	-0.109*	-0.126**	0.286**	1.000								
Meal	-0.070	0.045	-0.081	0.054	0.059	1.000							
Sleep	-0.073	-0.001	-0.092*	0.003	0.090*	0.572**	1.000						
Exercise	0.070	-0.131**	0.125**	0.041	0.001	-0.118**	-0.182**	1.000					
Internet	-0.120**	0.041	-0.064	-0.139**	-0.059	0.181**	0.201**	-0.083	1.000				
Anxiety	-0.042	0.078	-0.147**	0.048	0.120**	0.475**	0.514**	-0.190**	0.133**	1.000			
Depression	-0.074	0.035	-0.150**	0.052	0.152**	0.506**	0.571**	-0.156**	0.211**	0.798**	1.000		
Fear of COVID-19	-0.107*	0.099*	-0.153**	0.101*	0.011	0.232**	0.227**	-0.128**	0.055	0.392**	0.345**	1.000	
DT-FBD	0.304	0.169**	-0.028	0.030	0.094*	0.449**	0.412**	-0.083	0.111*	0.519**	0.564**	0.267**	1.000

Note. * p < 0.05. ** p < 0.01

Meal, frequency of irregularity of three meals in the last two weeks; Sleep, frequency of irregular sleep in the last two weeks; Exercise, average daily exercise time in the last two weeks; Internet, average daily Internet usage in the last two weeks; DT-FBD, the Diagnostic Tendency of Functional Bowel Disease

**Table 6:** Frequency of irregular diet, depression and anxiety levels as mediators of effect size

Affect	Point Estimate (95% CI)	Proportion, %
fear of COVID-19→DT-FBD	0.068 (0.066 − 0.029)	55.28
fear of COVID-19→depression→DT-FBD	0.041 (0.026–0.062)	33.34
fear of COVID-19→anxiety→meal→DT-FBD	0.004 (0.001–0.008)	3.25
fear of COVID-19→depression→meal→DT-FBD	0.009 (0.005–0.016)	7.3
Total	0.123	100

DT-FBD, the Diagnostic Tendency of Functional Bowel Disease; Meal, frequency of irregularity of three meals in the last two weeks

## Discussion

According to our analysis, students living under closed management measures at colleges and universities generally experienced greater fear of COVID-19 and exhibited more severe symptoms of anxiety and depression during the fourth stage of the pandemic in China. Allé et al. found that psychotic symptoms were higher in individuals with less social interaction, longer self-isolation, and smaller living spaces [30]. Students living in closed-management schools experienced social isolation (lacking contact with the outside world) and lived in small spaces for a long time (their activities were limited to their dormitories or schools, without other activity spaces), which we speculate may explain our findings. Universities in high-risk areas reported more frequent closure measures than those in medium- and low-risk areas. Logistic regression analysis revealed that students in high-risk areas were more likely to have more severe anxiety and depressive symptoms, which confirms our predictions to some extent. Dormitory relationships are also a concern as interpersonal relationships play an important role in mental health [31]. Students in closed-management dormitories were shown to be more intimate, have relatively higher interpersonal sensitivity and be more likely to have physical and mental problems [32].

Poor lifestyle habits are important risk factors for anxiety and depression. Four categories of lifestyle habits were included in this study: sleep, diet, exercise, and time spent using the internet. The higher the frequencies of irregular eating and sleep, the less time spent exercising, and the more time spent online in the last two weeks, the more severe the anxiety (all p < 0.05) and depressive symptoms were (all p < 0.05 but P = 0.117 for exercise). Healthy behavior, good sleep and a balanced diet can improve mental health [33–35]. The likelihood of disruption of healthy lifestyle habits is clearly elevated during closed management. Time using the internet was positively associated with the likelihood of anxiety and depression, similar to previous reports [36]. It is clear that people spend more time studying and working on the internet during closed management. In addition, depression and anxiety symptoms were also significantly positively correlation with fear of COVID-19, which we consider a direct effect of the pandemic on mental health.

In the present study, 49% of the students had a greater likelihood of being diagnosed with FBD. FBD is strongly associated with lifestyle habits and mental health [10, 17, 20]. Our findings indicated that poor eating and sleeping habits, more severe depressive and anxiety symptoms, and greater fear of COVID-19 were all risk factors for FBD. Based on relevant studies, we suggest that anxiety and depressive symptoms impact gastrointestinal symptoms mainly through (i) effects on the motor response and visceral sensitivity of the gastrointestinal system; (ii) interactions with the central nervous system and the gastrointestinal system, leading to dysregulation of the brain-gut axis [5, 33, 34, 37–42]; and (iii) influencing lifestyle habits such as diet [43–45]. Mönnikes et al. viewed stressful events as an essential factor in the aggravation of symptoms of functional gastrointestinal disorders, especially intestinal stress syndrome; this may explain the impact of fear of COVID (a mental health factor) on the likelihood of being diagnosed with FBD [46]. In addition, sex also influenced the likelihood of being diagnosed with FBD, which is consistent with previous findings [47, 48]. Sex differences in gastrointestinal dynamics and brain activation patterns in response to visceral stimulation have been reported, and higher visceral sensitivity and sex hormone levels in women may contribute to their higher risk of FBD [49, 50].

It is important to assess the specific mechanisms by which symptoms occur to select appropriate interventions to reduce their incidence and severity [51]. The mediation analysis in this study revealed the following associations among the included risk factors in college students: fear of COVID-19 increased the incidence and severity of depression and anxiety symptoms, while depression and anxiety symptoms increased the frequency of irregular eating, which in turn increased the likelihood of being diagnosed with FBD.

Therefore, we recommend that universities increase real-time monitoring of students’ mental health and improve the availability of psychological counseling and intervention services to help students better cope with stress and other risk factors under closed management measures. Furthermore, psychological disorders exerted a direct effect on gastrointestinal health. Some intermediate factors behind this effect that we did not examine in this study, such as relationships and living space (mentioned in the previous section), merit further exploration. Many studies have shown that healthy lifestyle habits benefit physical and mental health [35, 52–54]. In our study, healthy diet and sleep, an average of > 30 min of exercise per day, and less time spent on the internet were associated with reduced fear of COVID-19, milder anxiety and depression symptoms, and a lower likelihood of being diagnosed with FBD among college students. Thus, we suggest that colleges and universities take appropriate measures to help students improve poor lifestyle habits and protect the physical and mental health of college students during the pandemic.

This study has several strengths. First, it is the first study to assess the likelihood of being diagnosed with FBD and risk factors for FBD among college students during the epidemic. We developed a novel assessment scale, the DT-FBD, which can be quickly completed by college students in daily life and may provide a new reference for healthy lifestyle management. Second, despite changes in current prevention and control policies in China, our study can provide guidance for future health management during stressful events such as infectious disease outbreaks. Third, this study explored the influencing factors and interconnections through mediation analysis, which lays the foundation for further research.

The study also had some shortcomings, such as the small sample size, and potential bias in results. To persuade more people to participate in this experiment, the questionnaire contained fewer items; thus, it may not reflect the actual situation as accurately as the original. In addition, our custom-designed scale (the DT-FBD) can indicate only a greater likelihood of being diagnosed with FBD but cannot be used as for clinical diagnosis. There are few similar studies on gastrointestinal tract symptoms in college students, and there is a lack of reference data and related results for comparison. Finally, the cross-sectional design prevents evaluation of causality.

## Conclusion

The closed management of colleges and universities poses a threat to the psychological and gastrointestinal health of college students, which can be improved by healthy lifestyle habits. Therefore, we recommend that college students attempt to engage in healthy lifestyle habits and increase their physical exercise. Moreover, schools should strengthen real-time monitoring of students’ mental health and make appropriate interventions available. The findings may help to develop guidelines to mitigate the impact of stressful events (such as other infectious disease outbreaks) on physical and mental health among college students and provide a reference for the management of future stressful events.

## Electronic supplementary material

Below is the link to the electronic supplementary material.

Supplementary Material 1

## Data Availability

All data generated or analysed during this study are included in this published article [and its supplementary information files].

## Abbreviations

GAD-7: The 7-item Generalized Anxiety Disorder Scale
PHQ-9: The 9-item Patient Health Questionnaire
FCV-19S: The Fear of COVID-19 Scale
DT-FBD: The Diagnostic Tendency of Functional Bowel Disease Scale
FBD: Functional bowel disease
IBS: Irritable bowel syndrome
IQR: Interquartile range
CIs: Confidence intervals
